# On the Motion of Spikes: Turbulent-Like Neuronal Activity in the Human Basal Ganglia

**DOI:** 10.3389/fnhum.2018.00429

**Published:** 2018-10-24

**Authors:** Daniela Andres

**Affiliations:** Science and Technology School, National University of San Martin, Buenos Aires, Argentina

**Keywords:** structure function, Parkinson’s disease, neuronal activity, turbulence modeling, neuronal modeling, basal ganglia, complexity, non-linear dynamics

## Abstract

Neuronal signals are usually characterized in terms of their discharge rate, a description inadequate to account for the complex temporal organization of spike trains. Complex temporal properties, which are characteristic of neuronal systems, can only be described with the appropriate, complex mathematical tools. Here, I apply high order structure functions to the analysis of neuronal signals recorded from parkinsonian patients during functional neurosurgery, recovering multifractal properties. To achieve an accurate model of such multifractality is critical for understanding the basal ganglia, since other non-linear properties, such as entropy, depend on the fractal properties of complex systems. I propose a new approach to the study of neuronal signals: to study spiking activity in terms of the velocity of spikes, defining it as the inverse function of the instantaneous frequency. I introduce a neural field model that includes a non-linear gradient field, representing neuronal excitability, and a diffusive term to consider the physical properties of the electric field. Multifractality is present in the model for a range of diffusion coefficients, and multifractal temporal properties are mirrored into space. The model reproduces the behavior of human basal ganglia neurons and shows that it is like that of turbulent fluids. The results obtained from the model predict that passive electric properties of neuronal activity, including ephaptic coupling, are far more relevant to the human brain than what is usually considered: passive electric properties determine the temporal and spatial organization of neuronal activity in the neural tissue.

## Introduction

The basal ganglia are a circuit of densely interconnected subcortical nuclei, whose disease is related to human movement disorders (Obeso et al., 2008). Current models of the basal ganglia are partly successful in the prediction of neurophysiologic alterations occurring in movement disorders, including Parkinson’s disease. However, no current model allows to predict and control deep brain stimulation (DBS), one of the major therapeutic approaches to Parkinson’s disease (Montgomery, 2016). The lack of a description of the complex properties of basal ganglia neuronal activity might be one cause of this failure. The fundament of the classic model of pathophysiology of Parkinson’s disease lies on an excessive discharge rate of the output nuclei of the basal ganglia: the globus pallidus interna (GPi), and substantia nigra reticulata (SNr) (Albin et al., 1989; DeLong, 1990). In primates, GPi and SNr neurons fire in a tonic manner, keeping the motor thalamus inhibited, and momentary reductions of their discharge rate facilitate movement. In humans, movement onset and velocity are positively correlated to the rate of discharge of the motor thalamus, and negatively to the rate of discharge of the GPi and SNr (Utter and Basso, 2008). Although these observations seem to confirm the classic model, evidence obtained during functional neurosurgery in patients with Parkinson’s disease is contradictory, since high frequency DBS increases the rate of discharge of the GPi instead of lowering it (Anderson et al., 2003; Montgomery, 2006; Andres and Darbin, 2017). Applying microdialysis techniques it has been shown that clinically effective DBS increases cyclic guanosine monophosphate (cGMP) in the putamen and GPi, supporting an increase of activity in these nuclei (Galati et al., 2006; Stefani et al., 2011a,b). This suggests that other features of neuronal activity than the rate of discharge need to be taken into account to understand the origin of parkinsonian symptoms and their amelioration by DBS. In summary, the predictions of the classic model of the basal ganglia are inconsistent with the effects of DBS and ablative surgery, raising concerns on the validity of the rate of discharge as a measure of the state of the basal ganglia (Tang et al., 2005; Montgomery and Gale, 2008).

Non-linear time series analysis has inspired an alternative view. A growing body of evidence indicates that complex properties, in particular fractality, are crucial for the understanding of basal ganglia pathophysiology (Obeso et al., 2000; Zheng et al., 2005; Darbin et al., 2006; Rasouli et al., 2006; Lim et al., 2010; Andres et al., 2011a; Alam et al., 2015). Complex and non-linear temporal properties are present in basal ganglia spike trains from rodents, primates and patients with movement disorders (Li et al., 2008; Lim et al., 2010; Hohlefeld et al., 2012, 2015; Darbin et al., 2013). The most popular non-linear measure for the characterization of parkinsonian spike trains is entropy, which diminishes due to pharmacologic or stimulation treatment, and increases due to alertness (Lafreniere-Roula et al., 2010; Lim et al., 2010; Andres et al., 2014a; Alam et al., 2015; Darbin et al., 2015). Importantly, complex properties cannot be understood isolated, but they relate to each other. For instance, entropy can be estimated wrongly if the multifractality of a system is not considered (Costa et al., 1997; Lyra and Tsallis, 1998; Latora et al., 2000). Hence the need to find methods that can be used to study complexity at this level, and equations to reproduce it.

This paper investigates some similarities between the fractal organization of spike trains from parkinsonian neurons and turbulent fluids. It presents a descriptive study of human pathology and a simulation study. In previous work, I hypothesized that neurons produce signals with correlations on different scales (Andres et al., 2014b). This hypothesis gives a reason to analyze high order structure functions. Since structure functions of order M remove polynomials of order M-1, low order linear processes are progressively eliminated by the analysis, highlighting the effect of stationary, high order, complex dynamics (Simonetti et al., 1985). This method was developed initially to describe the behavior of turbulent fields in the inertial range, and was used successfully both in experimental and simulation studies (Kolmogorov, 1941; Benzi et al., 1993; Briscolini et al., 1994). Later structure functions were applied to the study of physiologic signals (Lin and Hughson, 2001; Nanni and Andres, 2017). Although the length of the time series analyzed must increase depending on the time lag studied, the study of structure functions is robust to short time series in comparison to other tools (Schulz-DuBois and Rehberg, 1981; Van de Water and Herweijer, 1999; Monin and Yaglom, 2013; Nanni and Andres, 2017). Time series of a length of thousands of data points have been analyzed with high order structure functions in different fields, and it was shown that this is a suitable method to look for non-linearity in the exponent function, i.e., to distinguish monofractality from multifractality (Yu et al., 2003; Huang et al., 2011). The paper is organized as follows. First it is shown that multifractality can be measured in neuronal signals from the GPi analyzing structure functions of increasing order. Then a neural field model is introduced inspired by Burgers’ equation, a well-known equation in the field of fluid dynamics (Burns et al., 1998). Previous evidence indicated that this kind of equations could be useful to study mathematical properties of neuronal activity, which is analyzed here (Andres et al., 2011b). The model is based on physical properties of neurons, and it captures essential features of neuronal activity of the human brain, reproducing multifractality as observed in the neuronal activity of the human, parkinsonian basal ganglia.

## Methods and Results

### Patients and Clinical Recordings

Six patients fulfilling the clinical criteria of the United Kingdom’s Parkinson’s Disease Society Brain Bank for idiopathic Parkinson’s (UK-PDS-BB) disease, Hoehn and Yahr (1967) IV, underwent stereotactic neurosurgery and were included in this study. All patients were on chronic treatment with L-DOPA, presented similar motor affectation, with severe dyskinesias and motor fluctuations, and fulfilled the Core Assessment Program for Surgical Interventional Therapies in Parkinson’s Disease (CAPSIT) criteria for inclusion in the surgery program (Defer et al., 1999). All patients signed informed consent prior to the procedure, previously reviewed and approved by the institutional ethics committee, and were without medication at the time of the surgery and during data acquisition. Age, gender, and other details are not shown to protect patients’ privacy.

In total, 22 GPi recordings obtained from 6 patients were analyzed. Details about the recording procedure have been already published elsewhere (Andres et al., 2011a). Briefly, microrecording, stimulation, and neurosurgical procedures were performed in patients awake, under local anesthesia. Surgical targets were planned employing magnetic resonance imaging (MRI) and using a Leksell stereotactic system (Series G, Elekta, Sweden). Microrecordings of neuronal activity were obtained only during the surgery, after the GPi was identified by an expert. Platinum/iridium (Pt/Ir 80/20%) microelectrodes with nominal impedance of 0.8–1.2 megohms (mTSPBN-LX1, FHC Inc.) were used. A differential amplifier with a built-in impedance meter (FHC IS-AM-00-01 Iso-Xcell 3 Amplifier) and an isolated stimulus generator (FHC IS-PL-06 Isolated Bipolar Pulsar Stimulator; FHC, Bowdoinham, ME, United States) were connected to a preamplifier remote probe mounted onto a motorized microdrive (FHC 65-00-1 Stepper Drive and ST-M0-00 TMS Controller), located near the electrode tip to minimize pickup of electrical noise. A dedicated acquisition system (1401plus, CED) was employed to amplify, and digitize the signal, filtering with second order 300–5,000 Hz bandpass and 50 Hz line notch analog filters. The sampling rate was 50 kHz. The total amplification including probe was set at gain ×10000 (checked with a built-in calibration signal of 1 mV at the beginning of each surgery).

### Signal Processing

Signals were processed off-line. To obtain single cell recordings, spikes were extracted from raw signals and separated into classes using wavelet analysis and clustering (Quiroga et al., 2004; Andres et al., 2014a). From these single cell data, time series of interspike intervals (ISI) were constructed. Time series had a mean length of 5668 ± 773 ISI (mean ± standard error of the mean, SEM). From these time series, the temporal structure function was computed as follows. An interval I(t) is defined as the *t*th ISI, which is used to calculate the increment ΔI(τ) = I(t + τ) - I(t), being τ the time lag, between 1 and 1000. The temporal structure function S_q_(τ) is

(1) $${\text{S}}_{\text{q}}(\tau )=\langle |\Delta \text{I(}\tau {\text{)|}}^{\text{q}}\rangle ,$$

where ⟨⋅⟩ is the statistical average, and q is the order of the structure function, an integer between 1 and 30. Next, S_q_ was smoothed by applying a running average over a 30 points window to obtain a smoothed structure function, ${\text{S}}_{\text{q}}^{*}$ (30 points shorter than S_q_). The behavior of interest here is the scaling behavior of ${\text{S}}_{\text{q}}^{*}$(τ). Given the general relationship

(2) $${\text{S}}_{\text{q}}^{*}(\tau )\sim \tau^{\zeta (q)},$$

the scaling exponent is a function, ζ_τ_(q), which defines the fractal properties of the signal under study. For random processes, ζ_τ_(q) = 0. In the case of monofractals the exponent function grows linearly, ζ_τ_(q) = qζ(1). If the signal is multifractal, ζ_τ_(q) is a non-linear function (Lin and Hughson, 2001). Note that different authors use different notations. The function that is called here ζ_τ_(q) is called τ(q) in Touchette’s work, according to whom the spectrum of singularities f(α) does not need to be concave (Touchette and Beck, 2006). A concave f(α) can be calculated as the Legendre transform of ζ_τ_(q), whereas a not concave f(α) cannot. In any case, a concave ζ_τ_(q) implies multifractality.

To find ζ_τ_(q), log(${\text{S}}_{\text{q}}^{*}$(τ)) vs. log(τ) was considered, and a linear (scaling) region was looked for. The scaling region was defined as the longest range of τ fitting a linear function with non-zero slope (ζ≠0), for at least 1 ≤ q ≤ 10. Linear regressions were considered acceptable if R^2^ ≥ 0.6, and regressions with regression coefficients smaller than that were discarded. A scaling region of the temporal structure function was found for a minimum range of 1 ≤ q ≤ 10 in every neuron. In 15 neurons, the fitting was sufficiently good for q up to 30 (q_max_ = 24 ± 2, mean ± SEM). The length of the scaling interval over τ was 79 ± 25; mean ± SEM.

**FIGURE 1:**
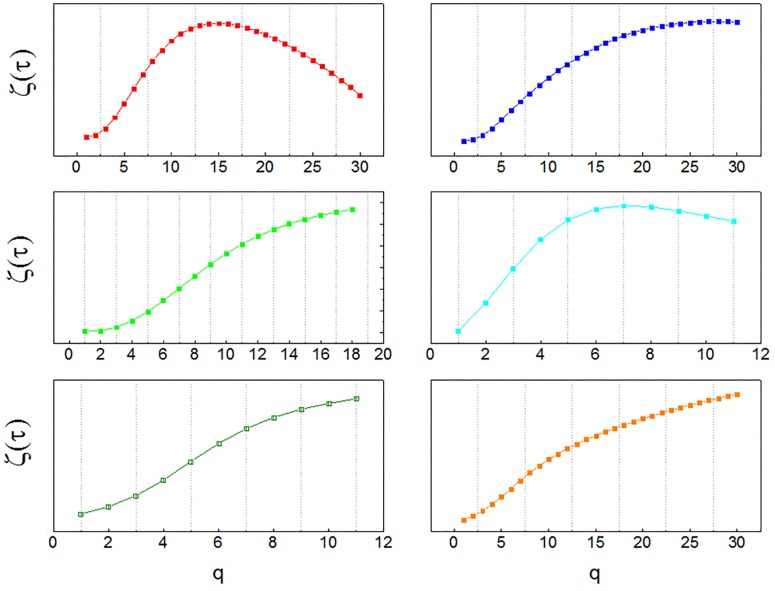
Multifractal spectra of human neurons. Temporal multifractal spectra of sample neuronal recordings, obtained from the globus pallidus interna (GPi) of patients with Parkinson’s disease with the structure function method. The non-linearity of ζ_τ_(q) indicates multifractality. The function ζ_τ_(q) is calculated from temporal structure functions of increasing order q, S_q_(τ). Since structure functions are built from time series of neuronal activity, the spectra indicate multifractal organization in the temporal domain.

Results for the analysis of neuronal recordings obtained from the patients with Parkinson’s disease are shown in Figure 1. A multifractal organization of spike trains was found in 82% of neurons, evidenced by non-linearity of the temporal exponent function (ζ_τ_(q)), obtained from structure functions of increasing order.

### Model and Simulations

To reproduce the behavior observed in the clinical data, a model is introduced that takes the form of a neural field equation, as follows:

(3) $$\frac{\partial \text{u}}{\partial \text{t}}+\text{u}\frac{\partial \text{u}}{\partial \text{x}}=\delta \frac{\partial^{2}\text{u}}{\partial {\text{x}}^{\text{2}}}+a\text{W,}$$

where u(x,t) represents the velocity of spikes (a function of time t and space x), δ is a diffusion coefficient and W is a stochastic drive with amplitude *a*.

The model describes neuronal activity in terms of the velocity of spikes. While spikes travel with constant velocity along axons, the same does not hold for neural tissue or neural networks at larger scales. Indeed, consider the following simplified situation. Take a single spike S that arrives to a neuronal soma whose membrane potential V is below the triggering threshold 𝜗. If this spike induces an excitation with an amplitude e in the neuron, such that the V + e > 𝜗, then the neuron will fire in response, which is equivalent to saying that the spike S has traveled through the neuron (a node of the network). Importantly, the condition V + e > 𝜗 can be satisfied for a range of potentials V and, since summation is a time-consuming mechanism, the spike triggered in response to the excitation e will be fired faster for membrane potentials that are closer to the threshold, giving place to a higher velocity of transmission u. Not only is velocity variable, but it is non-linearly so: the process of summation is typically a non-linear function of time, introducing non-linear variability in the profile of u. Even more, due to the close relation between the variables u (velocity of spikes) and f (neuronal frequency), it is expected that any non-linearity of u will be reflected on the time series of interspike intervals. The relation between both variables is simple: since u = Δx/Δt and f = 1/Δt, then u = f ⋅Δx. Consequently, for distances Δx = 1 the module of the velocity |u| has the same value than the frequency. Regarding the time intervals between spikes (interspike intervals I, ISI), they are equal to the inverse of the instantaneous frequency of discharge, and therefore |u| = 1/I.

Computer simulations were run with custom MATLAB and R code. The model was studied numerically in 1D. The discretization scheme selected was a Crank-Nicholson scheme, which is suitable for this kind of study (Kadalbajoo and Awasthi, 2006). The space steps and time steps were Δx = Δt = 0.001, and u(t) = 0 at the boundaries of the spatial domain. The initial condition considered was a random distribution of u(x) between -1 and 1, with a power spectrum obeying the scaling law P(ω(u)) = ω^β^, where β < 0. Following Hayot and Jayaprakash (1996), the same kind of colored noise W was used as a drive to the equation, with an amplitude a = 10^-6^. The temporal evolution of u(x) was studied for 1000 time and space steps and for a range of diffusion coefficients 10^-3^ < δ < 0.11, varying δ in increments of 0.02. To make the data obtained from the model more directly comparable to clinical data, the inverse of the absolute value of the velocity was considered, obtaining a variable equivalent to I(x,t). This variable $\text{I(x, t)}=\frac{1}{|\text{u|}}$ was analyzed applying the same algorithm that was described in point 2.2 for the analysis of clinical ISI time series to obtain a temporal structure function S_q_(τ) at fixed points in space. Finally, a spatial structure function S_q_(X) was computed in a similar manner, but considering adjacent points of u for fixed times. Scales between 1 and 50 were used to compute the structure functions both in space and time. A multifractal spectrum was looked for both in the space (ζ__X__(q)) and time domains (ζ_τ_(q)), by analyzing the behavior of S_q_(τ) ∼ τ^(q)^ and S_q_(X) ∼ X^(q)^ for q varying between 0 and 2 in steps of 0.1.

**FIGURE 2:**
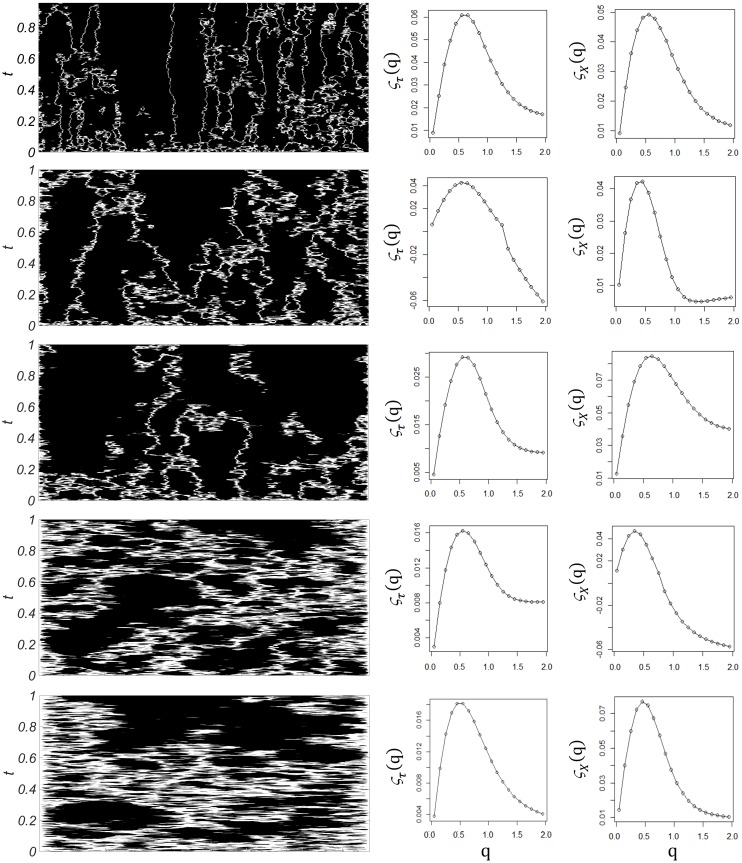
Computer simulations. Left column: Time evolution of the velocity of spikes, u(x,t), as the diffusion coefficient δ increases (from top to bottom), with time on the vertical axis and space on the horizontal axis. White areas represent the parts of the integration domain where the module of the velocity of spikes is below an arbitrary limit (10^8^), in opposition to black areas, where it is higher than this limit. As the diffusion coefficient increases, white areas are enlarged, as the total velocity diminishes across the integration domain. Middle column: Sample temporal multifractal spectra ζ_τ_(q) obtained from temporal structure functions of increasing order, at fixed spatial points. Non-linearity indicates temporal multifractality. Right column: Sample spatial multifractal spectra ζ_x_(q) obtained from spatial structure functions of increasing order, at fixed times. Non-linearity indicates spatial multifractality.

The diffusion coefficient is the critical parameter in the model. Figure 2 (left column) shows the results of computer simulations for increasing diffusion coefficient δ. At the white areas the module of the velocity of spikes (equivalent to frequency) is lowest, $\frac{1}{|\text{u(x, t)|}}\geq 10^{8}$, in opposition to the black areas, where it is highest. As the diffusion coefficient increases, white areas are enlarged, as the total area of high velocity diminishes. At the same time the whole integration domain becomes more homogeneous, with individual areas of high velocity becoming wider. This indicates that diffusion not only contributes to the dissipation of energy (lowering the global velocity of spikes transmission), but it also modifies the temporal and spatial organization of the neuronal activity across the neural field. Importantly, hallmarks of multifractality appear both in space and time for the whole range of δ investigated. The middle and right columns of Figure 2 show typical examples of the temporal and spatial multifractal spectra obtained from the model, ζ_τ_(q) and ζ__X__(q), respectively. Multifractality is characteristic of the model, with non-linear exponent functions in the spatial as well as temporal domains.

## Conclusion

In the neural field studied, complex geometrical properties arise from a combination of diffusive properties, a gradient field and a stochastic drive, in a manner similar to that of fluids undergoing turbulence. While this is an analogy, and any physical implications should be considered with care, some inferences might be interesting. For instance, the model provides a mechanism for the generation of the complex patterns observed in parkinsonian neuronal activity, i.e., that high-pass filtering of turbulent-like velocity signals produces intermittent bursts in non-linear systems with stochastic drive, such as the present one (Frisch and Morf, 1981). Here the stochastic drive can be interpreted as a high dimensional environmental input to the neural network, but the gradient field represents an intrinsic property of the network, i.e., neuronal excitability at a specific point of space, determining the change of the spikes’ velocity. Regarding the diffusive term, diffusive properties are a physical consequence of neuronal processes, given the diffusive nature of electric fields. Previous modeling work on the basal ganglia showed that a diffusion coefficient is a critical parameter for the control of information transmission and information deterioration in the parkinsonian GPi (Andres et al., 2014b). However, the role of these so-called passive electric properties of neuronal activity is not usually considered in neurophysiology studies, which more commonly focus on spiking activity solely. In the neural field introduced here: (1) multifractality of neuronal spike trains is related to diffusive properties, and (2) multifractality of temporal activity is reflected on the spatial domain. The model predicts that passive (diffusive) properties of neuronal activity determine the structure of temporal and spatial neuronal activity in the basal ganglia, and must be considered in the study and treatment of movement disorders.

## Ethics Statement

The experimental protocol was revised and approved by FLENI Ethics Committee, Buenos Aires, Argentina.

## Author Contributions

DA was responsible for the full content of this article.

## Conflict of Interest Statement

The author declares that the research was conducted in the absence of any commercial or financial relationships that could be construed as a potential conflict of interest.
